# Predicting Measles Outbreaks in the United States: Evaluation of Machine Learning Approaches

**DOI:** 10.2196/42832

**Published:** 2023-04-04

**Authors:** Boshu Ru, Stephanie Kujawski, Nelson Lee Afanador, Richard Baumgartner, Manjiri Pawaskar, Amar Das

**Affiliations:** 1 Merck & Co, Inc West Point, PA United States; 2 Merck & Co, Inc Rahway, NJ United States

**Keywords:** measles, measles outbreaks, measles epidemiology, machine learning, epidemiology, hybrid machine learning, infectious disease modeling, infectious disease outbreak prediction, unsupervised machine learning, supervised machine learning, infectious disease, model, predict, outbreak

## Abstract

**Background:**

Measles, a highly contagious viral infection, is resurging in the United States, driven by international importation and declining domestic vaccination coverage. Despite this resurgence, measles outbreaks are still rare events that are difficult to predict. Improved methods to predict outbreaks at the county level would facilitate the optimal allocation of public health resources.

**Objective:**

We aimed to validate and compare extreme gradient boosting (XGBoost) and logistic regression, 2 supervised learning approaches, to predict the US counties most likely to experience measles cases. We also aimed to assess the performance of hybrid versions of these models that incorporated additional predictors generated by 2 clustering algorithms, hierarchical density-based spatial clustering of applications with noise (HDBSCAN) and unsupervised random forest (uRF).

**Methods:**

We constructed a supervised machine learning model based on XGBoost and unsupervised models based on HDBSCAN and uRF. The unsupervised models were used to investigate clustering patterns among counties with measles outbreaks; these clustering data were also incorporated into hybrid XGBoost models as additional input variables. The machine learning models were then compared to logistic regression models with and without input from the unsupervised models.

**Results:**

Both HDBSCAN and uRF identified clusters that included a high percentage of counties with measles outbreaks. XGBoost and XGBoost hybrid models outperformed logistic regression and logistic regression hybrid models, with the area under the receiver operating curve values of 0.920-0.926 versus 0.900-0.908, the area under the precision-recall curve values of 0.522-0.532 versus 0.485-0.513, and *F*_2_ scores of 0.595-0.601 versus 0.385-0.426. Logistic regression or logistic regression hybrid models had higher sensitivity than XGBoost or XGBoost hybrid models (0.837-0.857 vs 0.704-0.735) but a lower positive predictive value (0.122-0.141 vs 0.340-0.367) and specificity (0.793-0.821 vs 0.952-0.958). The hybrid versions of the logistic regression and XGBoost models had slightly higher areas under the precision-recall curve, specificity, and positive predictive values than the respective models that did not include any unsupervised features.

**Conclusions:**

XGBoost provided more accurate predictions of measles cases at the county level compared with logistic regression. The threshold of prediction in this model can be adjusted to align with each county’s resources, priorities, and risk for measles. While clustering pattern data from unsupervised machine learning approaches improved some aspects of model performance in this imbalanced data set, the optimal approach for the integration of such approaches with supervised machine learning models requires further investigation.

## Introduction

Measles is a highly contagious viral infection that can cause serious acute illness, complications including pneumonia and encephalitis, and death [1]. A population immunity of ~95% by 5 years of age is required to disrupt transmission [2]. A vaccination program initiated in the 1960s led to the formal elimination of measles in the United States in 2000 [3]. However, measles has recently resurged in the United States, with notable peaks occurring in 2014 (n=667 cases), 2018 (n=375), and 2019 (n=1282) [4-8].

Despite this resurgence, measles outbreaks are still rare events that are difficult to predict. Known correlates of measles exposure and transmission include international importations, high population density, and low vaccination coverage [9-13]. These factors vary substantially between and within states and can be used to help predict the likelihood and impact of measles outbreaks [9-12,14-16]. However, few prior studies have used quantitative approaches to estimate the risk of measles outbreaks at the county level. One recent model used a multiplicative risk function of 4 factors—measles, mumps, and rubella vaccination coverage; county population; the volume of international air travel; and the incidence of measles at the origin points of incoming international flights—to predict 20 high-risk counties, of which 17 had at least 1 measles case in 2019, accounting for ~55% of 2019 measles cases [9]. However, the model used only 4 predictors and was not validated using outbreak data from other years, meaning that its accuracy was not independently assessed. Measles prediction models could be further improved by incorporating additional county-level predictors of measles outbreak risk. For example, socioeconomic and demographic variables such as race or ethnicity, education, income, urbanicity, and health insurance coverage have been shown to correlate with measles vaccination coverage, while factors such as household composition may affect measles transmission rates [17-19].

The identification and modeling of additional measles risk predictors may require unbiased algorithmic approaches [20,21]. However, traditional statistical approaches, such as logistic regression, may be limited by incorrect assumptions about linearly independent predictor variables (ie, the predictors for neighboring counties may not be independent but rather multicollinear) and the low incidence of measles in the United States, which creates a data imbalance where the outcome of interest is a very rare event.

Machine learning (ML) methods provide several potential solutions to the above limitations. Decision tree–based ML approaches such as the extreme gradient boosting (XGBoost) classification model are inherently neutral to multicollinearity; the training process chooses the most informative predictor at any given decision or prediction split point, rather than using all provided predictors as in logistic regression. Many ML algorithms also permit adjustments to the balance between majority and minority class instances in the training data set; this regularization of the model, also referred to as cost-sensitive training, allows the classification models to learn more information from rare observations and avoid overfitting on the majority negative class [22].

Hybrid ML approaches that combine complementary models have been reported to have higher accuracy or a better interpretation of results than standalone models [23-25]. Combining supervised models such as XGBoost and logistic regression with unsupervised learning may help to overcome the challenges of predicting measles cases, based on the assumption that unsupervised learning processes will extract patterns from data that can be used as a new set of features that are less prone to biases introduced by multicollinearity and imbalanced data [26].

The objective of this study is to validate and compare XGBoost and logistic regression, 2 supervised learning approaches that are commonly used on tabular data, to predict the US counties most likely to experience measles cases. We compared these models with hybrid ML approaches that extended the XGBoost and logistic regression models to include additional predictors generated by 2 clustering algorithms, hierarchical density-based spatial clustering of applications with noise (HDBSCAN) and unsupervised random forest (uRF).

## Methods

### Design

**Figure 1:**
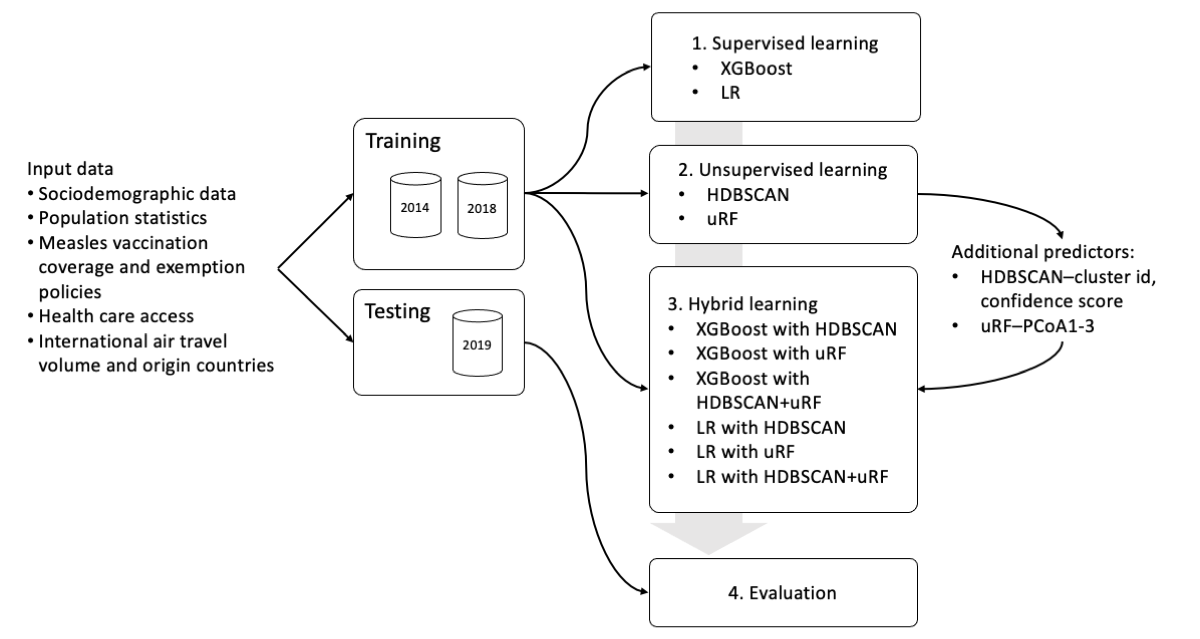
Study overview. HDBSCAN: hierarchical density-based spatial clustering of applications with noise; LR: logistic regression; PCoA: principal coordinate analysis; uRF: unsupervised random forest; XGBoost: extreme gradient boosting.

We used supervised (XGBoost and logistic regression) and unsupervised (HDBSCAN and uRF) ML analyses, as well as hybrid approaches that combined XGBoost and logistic regression with HDBSCAN, uRF, or both (Figure 1). All supervised and hybrid models were trained on input predictor variable data from 2014 to 2018 (training data set), with the cost-sensitive training option enabled. Predictor and outcome data from 2019 (testing data set) were used to evaluate all models.

### Data

The outcome of interest was the occurrence of ≥1 measles case at the county level. We performed a targeted search of published literature, state and local health department websites, and news articles to identify measles cases. We were able to identify information for 2895 counties in 2014, 2850 counties in 2018, and 2951 counties in 2019 and validate the county-level counts against published state-level counts [27]. Each county-year pair was considered 1 data point. Counties for which we could not validate measles case counts for each year were removed from the data set.

Variables relating to known and hypothesized predictors of measles outbreaks, based on the literature [9-12,14-16], were obtained from publicly available data sources at the county level (Multimedia Appendix 1) [28-42]. State- or metropolitan statistical area–level data were used as a proxy when county-level data were unavailable. Data were extracted from 1 year before the outcome year when possible, or else the closest possible prior year. Variables included sociodemographic data, population statistics, measles vaccination and exemption policies, health care access, and international air travel volume and origin countries (Multimedia Appendix 1).

We aggregated international air travel volume for each county and measles outbreak incidence at the origin of travel into a single score measuring the risk of exposure to measles via international air travel. The identification of trips from measles outbreak countries was based on the initial origin and final destination of travel using the same ticket [28]. The exposure scale was modeled using spatial diffusion, whereby international air travel passenger volumes to all US airports were proportionally distributed by population size to the county where the airport was located, the nearest neighbor counties, and the next-nearest neighbor counties, weighted by measles incidence at the travel origin and the county population [9].

The main models were run using all predictor variables, with a sensitivity analysis to account for multicollinearity. Multicollinearity between predictors was detected by the variance inflation factor and correlation matrices [43]. We hypothesized that eliminating predictor variables that were highly correlated would improve model performance and thus removed 10 predictor variables that were highly correlated to create a reduced version of the data set. The full list with summary statistics for each year and footnotes indicating variables removed in the reduced version is provided in Multimedia Appendix 2.

### Ethical Considerations

With the exception of the air travel data, all data were extracted from publicly available published literature, state and local health department websites, and news articles. All data were aggregated and deidentified, and, therefore, this study was exempt from institutional review board approval.

### Models

XGBoost is a gradient-boosting decision tree algorithm that is commonly used for classification and regression problems. The algorithm iteratively fits relatively simple models (typically small decision trees) to weighted versions of the training data. At each iteration, higher weights are assigned to data points that were misclassified by the model in the previous iteration; these are more likely to be from the minority class. Correctly predicting the minority class is thus rewarded more at each iteration. We magnified the weights assigned to data points in each iteration by the number of measles cases in the county + 1. This enabled the iterative training process to focus more on reducing classification errors for data points with more measles cases.

In standard logistic regression, classifying an event as a false positive (FP) or false negative (FN) carries the same penalty in the model. To address the challenge of imbalanced data, in which one of the dependent values occurs infrequently, we developed a weighted logistic regression approach that penalized the model more for an FN result. The weights were based on a cost-sensitive measure derived from the ratio between counties in the training data set with and without measles cases.

HDBSCAN is a density-based clustering algorithm that automatically optimizes cluster numbers and has the ability to work with noisy data [44,45]. We built an HDBSCAN model that maps each county-year observation into clusters using all predictor variables. HDBSCAN is a density-based clustering algorithm that automatically optimizes cluster numbers and has the ability to work with noisy data [44, 45]. We built an HDBSCAN model that maps each county-year observation into clusters using all predictor variables. A score measuring the algorithm’s confidence in assigning each observation to a cluster was also calculated. To investigate whether clustering results were informative for predicting measles cases, we compared the percentage of county-year pairs reporting measles outbreaks across the clusters. UMAP software was used to visualize clusters in multi-dimension space into two-dimension surface [46].

uRF combines many weak learners (individual decision trees) as a vehicle for variance and bias reduction [47,48]. Methods such as multidimensional scaling combined with hierarchical clustering are used to create a lower-dimensional representation of the observations. In this study, we fitted an uRF model to obtain the proximity matrix for each county-year’s predictor variable data in the training data set and then applied the model to project proximity matrices for the testing data set. Each county-year observation was then represented in 3 principal coordinates (PCoA.1-3), which we applied to the training and testing data sets to determine whether there were clustering patterns among counties reporting measles cases.

We also created 3 XGBoost and 3 logistic regression hybrid models that used outputs from HDBSCAN and uRF as additional features for making predictions. XGBoost and logistic regression with HDBSCAN models added cluster membership and confidence of clustering as new features; XGBoost and logistic regression with uRF models added PCoA.1-3; and XGBoost and logistic regression with HDBSCAN+uRF used both sets of new features. Data for 2014 and 2018 (5745 county-year pairs in total) were used as a training data set, and data for 2019 (2951 counties) were used as testing data set.

### Evaluation

The models were compared using evaluation metrics derived from the proportions of true positive (TP), FP, true negative (TN), and FN predictions. Sensitivity was defined as TP / (TP + FN), specificity as TN / (TN + FP), positive predictive value (PPV) as TP / (TP + FP), and the *F*_2_ score as (5 × PPV × sensitivity) / (4 × PPV + sensitivity). Given the highly infectious nature of measles, and thus the importance of sensitivity, we selected *F*_2_ over the more common *F*_1_ score, defined as (2 × PPV × sensitivity) / (PPV + sensitivity), to prioritize sensitivity over PPV.

The predicted class (positive or negative) of our models was determined at the threshold of 0.20 (eg, Y_prob_>0.20 → Y_pred_=1), which is smaller than the most commonly used value (0.50) due to data imbalance; adopting a lower threshold was expected to identify more counties vulnerable to measles outbreaks. Model prediction power was also measured using the area under the receiver operating curve (AUROC) and the area under the precision-recall curve (AUPRC), as suggested by previous studies on imbalanced data [22,49]. The AUROC values were calculated from plots of sensitivity against the FP rate across prediction thresholds and the AUPRC values from plots of PPV against sensitivity across prediction thresholds, with a perfect predictive model having an AUPRC and an AUROC of 1.0 and a coin-flip having an AUROC of 0.5 [49]. There is no fixed AUPRC value for random models; the baseline performance is commonly recognized as the percentage of positive class members, which was 3.1% for this study (proportion of US counties having ≥1 measles case in 2019) [49].

Data preprocessing and logistic regression modeling were conducted using SAS Studio release 3.8 (Basic Edition; SAS Institute, Inc). Python (version 3.6; distributed by Anaconda, Inc) with Pandas, Numpy, Scikit-learn, HDBSCAN, XGBoost, Matplotlib, UMAP libraries, and R (version 3.6.3; The R Foundation) with STATS package were used to build the XGBoost, HDBSCAN, and uRF models.

## Results

### Measles Cases

We were able to identify counties for 635/667 (95.2%) of Centers for Disease Control and Prevention–reported US measles cases in 2014, 366/375 (97.6%) of 2018 cases, and 1247/1287 (96.9%) of 2019 cases. In 2014, 81 of the 3143 (2.6%) counties in the United States had ≥1 measles case, while 64 (2.0%) had ≥1 measles case in 2018 and 98 (3.1%) in 2019.

### Unsupervised Machine Learning

**Figure 2:**
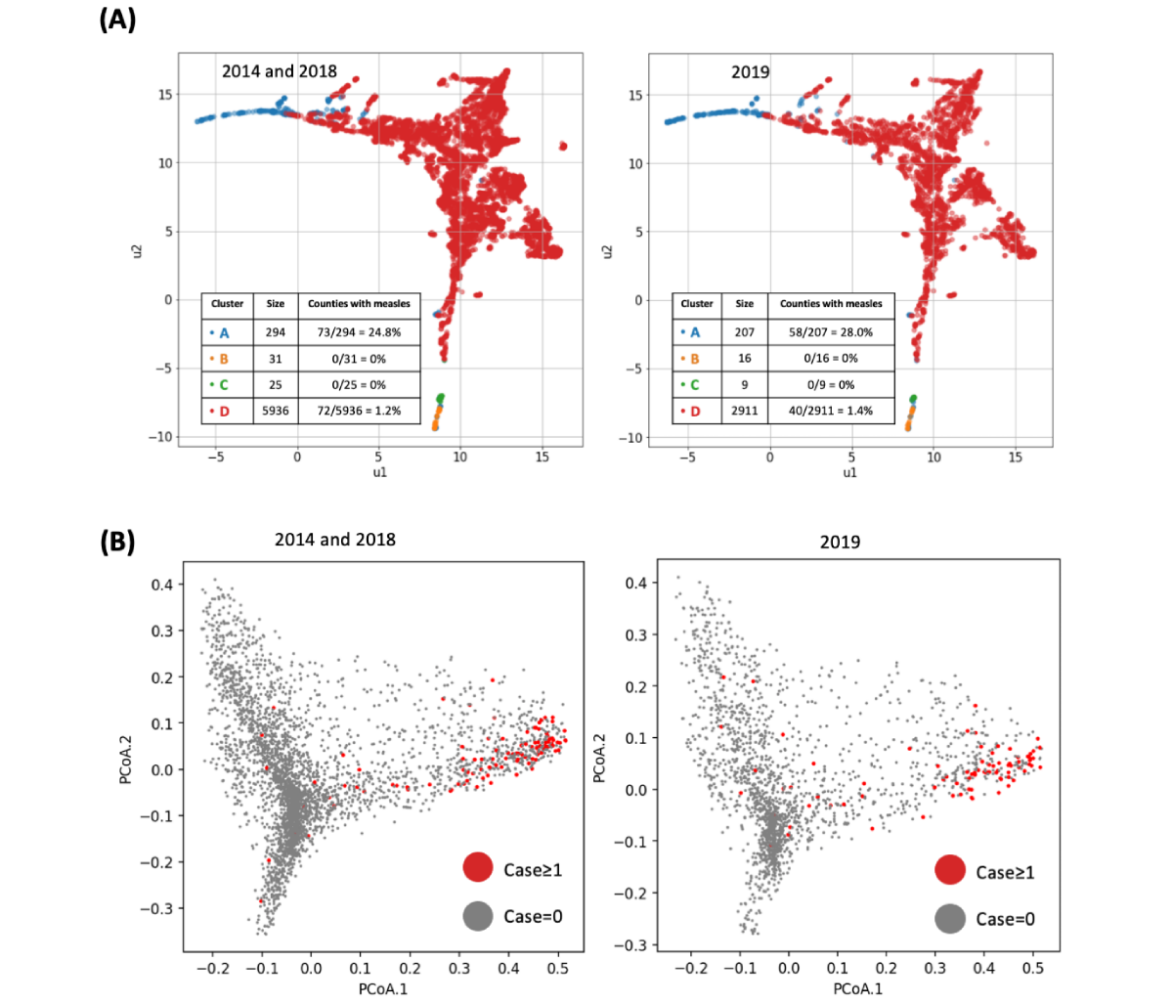
Unsupervised learning results. (A) HDBSCAN-identified clusters, color-coded by cluster size and percentage of counties reporting measles cases. (B) Visualization of counties with and without measles cases by 2 of 3 uRF-generated principal coordinates. HDBSCAN: hierarchical density-based spatial clustering of applications with noise; PCoA: principal coordinate analysis; uRF: unsupervised random forest.

The HDBSCAN model identified 4 clusters in the training data sets using all predictor variables (Figure 2A). The number of counties in clusters A and D with ≥1 measles case was 73/294 (24.8%) and 72/5936 (1.2%), respectively, while no counties with measles cases were found in clusters B or C. When applying the HDBSCAN clustering model to the testing data set, the measles cases also appeared only in clusters A and D, with frequencies of 58/207 (28%) and 40/2911 (1.4%), respectively.

The first and second PCoA derived by uRF for each county in the training and testing data sets were plotted using all predictor variables (Figure 2B). The observed clustering effects of counties with measles cases in the training and testing data sets were between 0.3 and 0.5 in the axis of PCoA.1 and between −0.05 and 1.5 for PCoA.2. These ranges are meaningful in that they reflect a similar projection of dissimilarities in both the training and testing data sets.

### Evaluation of Prediction Models

**Figure 3:**
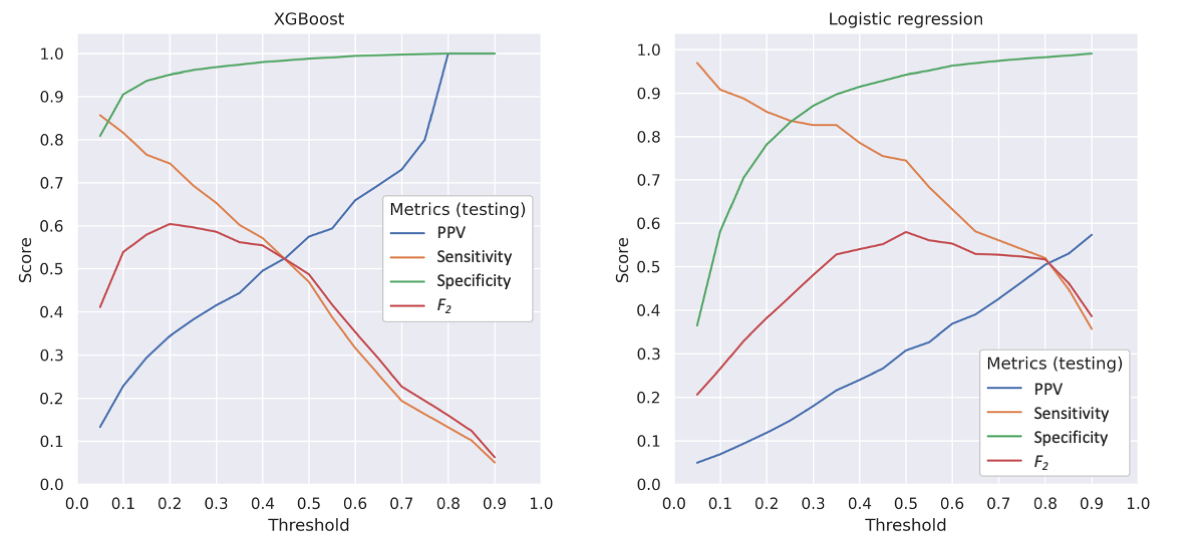
Comparative model performance at different prediction thresholds. *F*_2_ score = (5 × PPV × sensitivity) / (4 × PPV + sensitivity). PPV: positive predictive value; XGBoost: extreme gradient boosting.

The performance of all models at a prediction threshold of 0.20 is summarized in Table 1. The XGBoost and XGBoost hybrid models achieved higher AUROC and AUPRC scores than the logistic regression and logistic regression hybrid models (AUROC 0.920-0.926 vs 0.900-0.908; AUPRC 0.522-0.532 vs 0.485-0.513). All AUPRC values were considered high when compared with the low percentage of US counties reporting ≥1 measles case in 2019 (3.1%). At the threshold of 0.20, the hybrid models of XGBoost with HDBSCAN and uRF and XGBoost with uRF achieved the highest PPVs (0.367). Logistic regression with HDBSCAN and uRF features and logistic regression with uRF features produced the highest sensitivity (0.857), but the corresponding PPVs (0.141 and 0.139, respectively) were lower than those of the XGBoost and XGBoost hybrid models (0.340-0.367). XGBoost and XGBoost hybrid models had higher specificity (0.952-0.958) and *F*_2_ (0.595-0.601) than logistic regression and logistic regression hybrid models (0.793-0.821 and 0.385-0.426, respectively). For both XGBoost and logistic regression, the overall differences in performance measures between the original and hybrid versions of the same model were relatively small. The performance of all the models at a range of prediction thresholds between 0.0 and 1.0 is depicted in Figure 3.

**Table 1:** Performance of models predicting US counties with ≥1 measles case in 2019.

Model			PPV^a^		Sensitivity		Specificity		*F*_2_^b^		AUROC^c^		AUPRC^d^
**All variables**													
	XGBoost^e^	0.348		0.735		0.953		0.601		0.926		0.522	
	XGBoost with HDBSCAN^f^	0.340		0.724		0.952		0.591		0.924		0.525	
	XGBoost with uRF^g^	0.367		0.704		0.958		0.595		0.920		0.524	
	XGBoost with HDBSCAN+uRF	0.367		0.704		0.958		0.595		0.922		0.532	
	LR^h^	0.122		0.837		0.793		0.385		0.900		0.485	
	LR with HDBSCAN	0.125		0.837		0.798		0.391		0.900		0.497	
	LR with uRF	0.139		0.857		0.818		0.422		0.908		0.512	
	LR with HDBSCAN+uRF	0.141		0.857		0.821		0.426		0.907		0.513	
**Reduced data set**													
	XGBoost	0.333		0.724		0.950		0.587		0.931		0.525	
	XGBoost with HDBSCAN	0.340		0.735		0.951		0.596		0.930		0.519	
	XGBoost with uRF	0.335		0.724		0.951		0.588		0.924		0.515	
	XGBoost with HDBSCAN+uRF	0.326		0.735		0.948		0.587		0.927		0.515	
	LR	0.087		0.796		0.715		0.304		0.844		0.368	
	LR with HDBSCAN	0.096		0.867		0.720		0.333		0.894		0.402	
	LR with uRF	0.121		0.878		0.781		0.390		0.898		0.403	
	LR with HDBSCAN+uRF	0.119		0.867		0.779		0.384		0.902		0.433	

^a^PPV: positive predictive value.

^b^*F*_2_ score = (5 × PPV × sensitivity) / (4 × PPV + sensitivity).

^c^AUROC: area under the receiver operating curve.

^d^AUPRC: area under the precision-recall curve.

^e^XGBoost: extreme gradient boosting.

^f^HDBSCAN: hierarchical density-based spatial clustering of applications with noise.

^g^uRF: unsupervised random forest.

^h^LR: logistic regression.

As a sensitivity analysis, we also evaluated the performance of models trained on the reduced variable data set (Table 1). The XGBoost and XGBoost hybrid models outperformed the logistic regression and logistic regression hybrid models on this data set in terms of AUPRC (0.515-0.525 vs 0.368-0.433) and AUROC (0.924-0.931 vs 0.844-0.902) but had lower sensitivity (0.724-0.735 vs 0.796-0.878). The PPV, sensitivity, specificity, and *F*_2_ scores at a prediction threshold of 0.20 were very similar among the original and hybrid models of the same type, for both XGBoost and logistic regression. The performance of logistic regression and its hybrid models was more impacted by removing the correlated predictor variables, with lower AUROC and AUPRC scores than for the corresponding models using the full data set (0.844-0.902 vs 0.900-0.908 and 0.368-0.433 vs 0.485-0.513, respectively). In contrast, the performance of XGBoost and its hybrid models was similar between the 2 data sets.

## Discussion

This work developed supervised and hybrid ML models to identify US counties at risk of measles cases and compared them with predictions made using logistic regression. To our knowledge, this study is the first to determine the absolute risk of a county having a measles outbreak using ML approaches. This model is an improvement over the previous work done in this area, as it takes into account a comprehensive list of predictors that are associated with measles outbreaks to further improve the predictions.

Two different types of the unsupervised model could identify clusters or groups of counties that had ≥1 measles case. In the supervised learning analysis, all models achieved very high prediction scores for future measles outbreaks as measured by AUROC and AUPRC, with XGBoost and XGBoost hybrid models outperforming logistic regression and logistic regression hybrid models. Adding clustering results and principal coordinates from unsupervised learning models as additional predictors did not improve all performance metrics of XGBoost models; in contrast, adding these features improved all performance metrics of the logistic regression models by small margins. The optimal way to incorporate information from HDBSCAN, uRF, or other unsupervised clustering algorithms into prediction models remains an open question. One potential direction is to develop predictive models tailored to clusters of counties that were identified through unsupervised learning methods. We also found that removing 10 correlated predictors with high variance inflation factors did not improve model performance in this study; however, models with a reduced number of variables may provide more interpretable results and prove more practical for public health implementation by streamlining the data collection process. It is also worth mentioning that we presented evaluation metrics as point estimates instead of constructing approximate CIs by the bootstrapping or jackknife approaches, as is used in some research, because our models produced similar performance metrics, especially for AUROC and AUPRC, and comparing their rank and point estimates of scores was, therefore, sufficient [50].

In this study, we selected 0.20 as the threshold to calculate PPV, sensitivity, specificity, and *F*_2_. This was a subjective decision based on the rarity of measles outbreaks. The threshold can be adjusted depending on decision makers’ tolerance for FP and FN results; for example, counties with fewer resources may need to implement higher thresholds. A dedicated cost-utility model that anchors changes in costs and mortality to FP and FN rates can also be built and empirically evaluated in the future to guide threshold selection [51].

This study is subject to several limitations. We were unable to identify the affected county for a small proportion of measles cases, which may impact prediction accuracy. County-level data on vaccination coverage and exemption rates were not available for all counties, and metropolitan statistical area- or state-level data may not necessarily be good proxies. Some predictor variables were included based on the association between vaccine hesitancy and individual-level variables; including these variables at the county level may have introduced an atomistic fallacy [52]. Further, we only included 3 distinct years of data in the study; adding more years of data (when they become available) may improve the generalizability of the results. Finally, a spatial diffusion model was used to estimate the final destination counties of travelers after arrival at the destination airport, but we did not account for the risk of spreading via domestic air travel or other major long-distance domestic travel routes.

The COVID-19 pandemic has affected the volume and pattern of domestic and international air traffic and has negatively impacted the on-time administration of routine childhood vaccinations in the United States [53-55]. In the United States, the pandemic may have also increased hesitancy related to vaccines and altered the demographic patterns of this hesitancy [56]. However, the long-term impact of the pandemic on measles importation and the rates and patterns of vaccination coverage are not yet known. Predictive models of measles outbreak risk may therefore have to be adjusted before their application to years after 2019.

In conclusion, XGBoost outperformed logistic regression in predicting the US counties at risk of measles cases. Unsupervised learning models also identified clustering patterns for counties with measles cases, and these features helped to improve the PPVs of both XGBoost and logistic regression. Additional work on developing hybrid models that incorporate unsupervised ML methods may lead to further optimization of outbreak prediction.

## Appendix

Multimedia Appendix 1 Data sources.

Multimedia Appendix 2 Summary statistics for predictor variables of interest.

## Abbreviations

AUPRC: area under the precision-recall curve
AUROC: area under the receiver operating curve
FN: false negative
FP: false positive
HDBSCAN: hierarchical density-based spatial clustering of applications with noise
ML: machine learning
PCoA: principal coordinate analysis
PPV: positive predictive value
TN: true negative
TP: true positive
uRF: unsupervised random forest
XGBoost: extreme gradient boosting

## Data Availability

The air travel data were commercially licensed from Airport Strategy and Marketing Ltd [28]. Other data were extracted from published literature, state and local health department websites, and news articles, with data sources provided in Multimedia Appendices 1 and 2. Data sharing is not applicable to this paper as no data sets were generated during this study.
