# Supplementary material for: Predicting Measles Outbreaks in the United States: Evaluation of Machine Learning Approaches
Source: JMIR Form Res. 2023 Apr 4;7:e42832. doi: 10.2196/42832 (PMC10131820; doi:10.2196/42832)
Supplement: Multimedia Appendix 1 [file formative_v7i1e42832_app1.docx]

| **Variable** | **Data source** | **Geographic level** |
| --- | --- | --- |
| **Vaccination coverage** | | |
| Measles vaccination coverage | Publicly available data from state/local health departments; CDC [[43](#_ENREF_43), [44](#_ENREF_44)] | State or county |
| State policy on medical exemptions | State laws | State |
| State policy on non-medical exemptions | *ibid.* | State |
| **Vaccine-hesitant groups associated with previous measles outbreaks** | | |
| Amish population (12 largest US settlements) | Young Center for Anabaptist and Pietist Studies at Elizabethtown College [[45](#_ENREF_45)] | County |
| Church of Christ Scientists congregations | Association of Religion Data Archives [[46](#_ENREF_46)] | County |
| Orthodox Jewish congregations | *ibid.* | County |
| Somali population | US Census American Community Survey [[47](#_ENREF_47)] | County |
| **Population statistics** | | |
| Population density | CDC Social Vulnerability Index [[48](#_ENREF_48)] and US Census [[49](#_ENREF_49)] | County |
| County population | US Census [[49](#_ENREF_49)] | County |
| Percent urban | *ibid.* | County |
| Population age distribution (in 5-year categories) | *ibid.* | County |
| Percent by race/ethnicity | *ibid.* | County |
| Percent speaking English less than “well” | US Census American Community Survey [[47](#_ENREF_47)] | County |
| **Socioeconomic status** | | |
| Percent below poverty level | US Census Small Area Income and Poverty Estimates [[50](#_ENREF_50)] | County |
| Percent unemployment | US Bureau of Labor Statistics [[51](#_ENREF_51)] | County |
| Per capita income | US Bureau of Economic Analysis [[52](#_ENREF_52)] | County |
| Percent without a high school graduate diploma | US Census American Community Survey [[47](#_ENREF_47)] | County |
| **Housing and household composition** | | |
| Percent single-parent household | US Census American Community Survey [[47](#_ENREF_47)] | County |
| Percent crowded households ^a^ | *ibid.* | County |
| **Access to healthcare** | | |
| Percent uninsured | US Census Small Area Health Insurance Estimates Program [[53](#_ENREF_53)] | County |
| Percent with a usual source of care | CDC Behavioral Risk Factor Surveillance System [[54](#_ENREF_54)] | Select metropolitan statistical areas; State |
| **Exposure to measles via international air travel** | | |
| International air travel volume to county | MIDT data, Airport Strategy and Marketing Ltd (ASM) [[28](#_ENREF_28)] | Airport level |
| Measles outbreak incidence at origin of travel | WHO Measles Surveillance Data [[55](#_ENREF_55)] and UN Population Estimates [[56](#_ENREF_56)] | N/A |

^a^ Defined as more people than rooms.
