# Supplementary material for: Predicting Measles Outbreaks in the United States: Evaluation of Machine Learning Approaches
Source: JMIR Form Res. 2023 Apr 4;7:e42832. doi: 10.2196/42832 (PMC10131820; doi:10.2196/42832)
Supplement: Multimedia Appendix 2 [file formative_v7i1e42832_app2.docx]

| **Variable** | **Year** | | | | |
| --- | --- | --- | --- | --- | --- |
|  | **2014** | **2018** | | **2019** | |
| **Continuous variables: mean (IQR)** |  |  | |  | |
| County population | 100, 928 | 103,793 | | 104,335 | |
|  | (11,020, 67,434) | (10,907, 67,878) | | (10,923, 68,022) | |
| Population density (people / mile^2^) ^a^ | 323.46 | 330.02 | | 330.73 | |
|  | (16.86, 115.63) | (16.51, 117.01) | | (16.56, 117.62) | |
| Personal income per capita (USD / year) ^a^ | 38,838 | 42,133 | | 44,323 | |
|  | (31,748, 43,136) | (35129, 46416) | | (36,730, 48,887) | |
| Unemployment (%) | 7.4 (5.5, 9.0) | 4.6 (3.5, 5.3) | | 4.1 (3.1, 4.8) | |
| Education ≤ high school (%) | 50.3 (43.2, 58.2) | 48.2 (40.9, 56.0) | | 47.7 (40.3, 55.6) | |
| Speak English less than “well” (%) ^a^ | 3.5 (0.8, 3.9) | 3.4 (0.8, 4.0) | | 3.4 (0.8, 3.9) | |
| Urban (%) ^a^ | 41.4 (11.7, 66.7) | 41.4 (11.7, 66.7) | | 41.4 (11.7, 66.7) | |
| Crowded households (%) ^b^ | 2.4 (1.2, 2.8) | 2.4 (1.2, 2.9) | | 2.4 (1.2, 2.9) | |
| Income below poverty level (%) | 17.2 (12.5, 20.9) | 15.4 (10.9, 18.4) | | 15.2 (10.8, 18.3) | |
| Single-parent households (%) ^c^ | 8.8 (7.0, 10.2) | 8.4 (6.7, 9.9) | | 8.3 (6.6, 9.8) | |
| Population <10 years of age (%) | 12.5 (11.1, 13.6) | 12.12 (10.9, 13.3) | | 12.0 (10.7, 13.2) | |
| MMR vaccination coverage (%) | 93.1 (91.4, 96.8) | 92.2 (91.7, 97.1) | | 94.3 (92.6, 96.7) | |
| Usual source of healthcare ^a^ | 74.8 (71.2, 78.6) | 75.6 (72.7, 79.3) | | 75.1 (72.1, 78.9) | |
| Uninsured (%) | 17.6 (13.6, 20.9) | 11.5 (7.4, 14.5) | | 11.5 (7.5, 14.6) | |
| Race / ethnicity (%) |  |  | |  | |
| Asian | 1.3 (0.4, 1.1) | 1.5 (0.4, 1.3) | | 1.5 (0.4, 1.3) | |
| Black | 8.9 (0.6, 10.0) | 9.0 (0.7, 10.2) | | 9.0 (0.7, 10.2) | |
| Hispanic | 8.8 (2.0, 8.8) | 9.5 (2.3, 9.7) | | 9.7 (2.4, 10.0) | |
| Native American, Alaska Native, or Native Hawaiian / Pacific Islander | 2 (0.3, 0.8) | 2.0 (0.3, 0.9) | | 2.1 (0.3, 0.9) | |
| White | 77.4 (66.0, 93.5) | 76.4 (64.6, 92.6) | | 76.1 (64.4, 92.3) | |
| ≥2 races | 1.6 (01.0, 1.8) | 1.8 (1.1, 2.0) | | 1.8 (1.2, 2.0) | |
| Religious communities |  |  | |  | |
| Church of Christ Scientist (congregations / county) ^a^ | 0.4 (0.0, 0.0) | 0.4 (0.0, 0.0) | | 0.4 (0.0, 0.0) | |
| Orthodox Judaism (adherents / 1,000 population) ^a^ | 0.4 (0.0, 0.0) | 0.4 (0.0, 0.0) | | 0.4 (0.0, 0.0) | |
| Risk of exposure to measles via international air travel ^d^ | 0.3 (0.0, 0.0) | 0.2 (0.0, 0.0) | | 1.0 (0.0, 0.1) | |
| **Binary variables: n (%)** |  | |  | |  |
| Amish population ^a^ | 12 (0.4) | | 12 (0.4) | | 12 (0.4) |
| Somali population ^a^ | 104 (3.3) | | 104 (3.3) | | 104 (3.3) |
| Philosophical exemptions allowed by State ^a^ | 1,282 (40.8) | | 1,224 (38.9) | | 1,224 (38.9) |
| Religious exemptions allowed by State | 2,932 (93.3) | | 2,932 (93.3) | | 2, 932 (93.3) |

IQR, interquartile range; MMR, measles, mumps, and rubella

^a^ Variable excluded from reduced dataset analysis.

^b^ Defined as more people than rooms.

^c^ With child(ren) <18 years of age.

^d^ Aggregated risk of exposure to measles via international air travel aggregated by applying a spatial diffusion model.
